# The forecast of COVID-19 spread risk at the county level

**DOI:** 10.1186/s40537-021-00491-1

**Published:** 2021-07-07

**Authors:** Murtadha D. Hssayeni, Arjuna Chala, Roger Dev, Lili Xu, Jesse Shaw, Borko Furht, Behnaz Ghoraani

**Affiliations:** 1 Department of Computer and Electrical Engineering and Computer Science, Florida Atlantic University, Boca Raton, FL 33431 USA; 2 LexisNexis Risk Solution, Alpharetta, GA USA

**Keywords:** COVID-19 Forecast, Deep learning, Mobility, County demographics

## Abstract

The early detection of the coronavirus disease 2019 (COVID-19) outbreak is important to save people’s lives and restart the economy quickly and safely. People’s social behavior, reflected in their mobility data, plays a major role in spreading the disease. Therefore, we used the daily mobility data aggregated at the county level beside COVID-19 statistics and demographic information for short-term forecasting of COVID-19 outbreaks in the United States. The daily data are fed to a deep learning model based on Long Short-Term Memory (LSTM) to predict the accumulated number of COVID-19 cases in the next two weeks. A significant average correlation was achieved (*r*=0.83 (*p = 0.005*)) between the model predicted and actual accumulated cases in the interval from August 1, 2020 until January 22, 2021. The model predictions had *r* > 0.7 for 87% of the counties across the United States. A lower correlation was reported for the counties with total cases of <1000 during the test interval. The average mean absolute error (MAE) was 605.4 and decreased with a decrease in the total number of cases during the testing interval. The model was able to capture the effect of government responses on COVID-19 cases. Also, it was able to capture the effect of age demographics on the COVID-19 spread. It showed that the average daily cases decreased with a decrease in the retiree percentage and increased with an increase in the young percentage. Lessons learned from this study not only can help with managing the COVID-19 pandemic but also can help with early and effective management of possible future pandemics. The code used for this study was made publicly available on https://github.com/Murtadha44/covid-19-spread-risk.

**Supplementary Information:**

The online version contains supplementary material available at 10.1186/s40537-021-00491-1.

## Introduction

With the reopening of the world economy, one of the critical issues about the coronavirus disease 2019 (COVID-19) is the delay in the outbreak detection [1]. This delay may leave the health care facilities unprepared and may result in closing the economy again. The main reason for the outbreak detection delay is the delay in testing, the lack of information about how COVID-19 is spreading, and how people behave in this pandemic. Some places, such as restaurants and supermarkets, may not follow proper cleaning and disinfecting protocols or other government guidelines to prevent the spread of the disease. Also, some patients with COVID-19 are asymptomatic and may remain unidentified. However, they still spread the disease by direct contact or by their secretions in public places, increasing the disease reproduction rate [2].

There are two general epidemiological approaches to model the spread of the virus: mechanistic and forecasting [3]. The Mechanistic models mathematically formulate disease transmission by dividing the population into compartments such as susceptible, infectious, and recovered and working out a function of time for each compartment. One commonly used mechanistic model is the Susceptible-Exposed-Infectious-Recovered model (SEIR) [4]. This approach is known to be effective for long-term predictions but less effective for predicting the resurgence of the virus. Also, these models do not consider social behavior, which is essential to COVID-19 rate prediction. In addition, these models do not capture the effect of asymptomatic patients in the virus spread. It is known that about 40% to 45% of COVID-19 infections are asymptomatic and even continue the virus transmission for a more extended period than the symptomatic patients [5]. Forecasting models are statistical approaches trained for outbreak detection using prior data and dynamic social behavior such as Auto-Regressive Integrated Moving Average (ARIMA). These models built on the recent advances in machine learning and deep learning algorithms integrate the non-linear impact of social behavior to develop effective models for the early detection of infectious diseases [6, 7]. One example of such machine learning models is Long Short-Term Memory (LSTM), a deep, data-driven model [8], which has shown to outperform well-known ARIMA and Nonlinear Autoregression Neural Network (NARNN) models in 14-day predictions of COVID-19 cases in eight European countries in the work of Kırbas et al. [9]. These data-driven models can learn from the history of the disease. For example, they can use the mobility data (i.e., transportation and walking), which provides a near-real-time change in movement patterns, to learn the effect of social behavior on the reproduction rate. An increase in mobility shows an increase in the interaction between people, especially in areas with high population density. Therefore, feeding the mobility data to epidemiological forecasting models can help not only estimate COVID-19 growth but also evaluate the effects of government policies on COVID-19 spread [10]. They also can capture the impact of the asymptomatic patients on the outbreak when forecasting the virus spread [11].

In this paper, we utilize a deep learning model to predict the accumulated new COVID-19 cases. We hypothesize that an advanced deep learning model that can learn from the data patterns of COVID-19 statistics combined with demographics and the social behavior quantified by the mobility data can effectively predict the accumulated new COVID-19 cases. For this purpose, we developed our COVID-19 predictive model based on the US county data and predicted the accumulated cases in two coming weeks. Specifically, we use county-level demographics, COVID-19 statistics, and the driving-mobility data collected by Apple Maps App to train an LSTM deep learning model. The rationale for the prediction in two weeks is that COVID-19 symptoms may appear 2 to 14 days after the initial exposure according to the Center for Disease Control and Prevention (CDC) [12], so it is essential to know the short-term estimate of the infected people in two weeks. The prediction is at the county level to account for the influence of low-level local policies and provide better forecasting quality to support the nation and state forecasts. For example, short-term predictions of the accumulated cases can be used to plan and decide whether a lockdown is necessary during the holidays.

The paper is organized as follows. First, we described the current state-of-the-art methods, their limitations, and our contribution in "Related work" section. Next, an explanation of the dataset used in our research was provided in "Dataset" section. Then, we provided the details about the developed deep learning model in Methods section. Finally, "Results and discussion" section reported the evaluation metrics, results, and analysis, and the paper was concluded in "Conclusions" section.

## Related work

There is a large body of research toward fighting COVID-19 in different fields. Some focus on diagnosis using gene expression and X-ray images [7, 13, 14] or provide emotion care based on textual analysis [15]. Others concentrate on predicting protein structure, drug development [16] or forecasting COVID-19 cases and death [7, 17–19].

The research toward COVID-19 forecast using the mobility data has been limited to a county [20, 21], state, or metropolitan-area level [22–26]. Chang et al. integrated the mobility data with an SEIR model to forecast the COVID-19 spread in the 10 largest metropolitan cities in the US [22]. They used location data from mobile applications provided by SafeGraph company. Aleta et al. also integrated mobility data from mobile devices and demographic data with a mechanistic model to forecast COVID-19 spread in Boston metropolitan area.

To coordinate the forecasting of mortality and incident cases, CDC initiated the COVID-19 Forecast Hub in April 2020 [24, 25, 27]. Several modeling teams have contributed to the hub for forecasting mortality and incident cases at the nation and state level. Rodriguez et al. contributed using a framework based on deep neural network for providing forecast uncertainty [23].

At the county level, Kapoor et al. developed a Graph Neural Network to forecast only the next-day COVID-19 cases [28]. Next-day forecasting is highly correlated with the previous day which makes it less critical. In another work, Adiga et al. [29] developed a Bayesian ensemble of variety of models (e.g. Auto Regressive, SEIR, LSTM etc.) to forecast the weekly accumulated cases 1 to 4 weeks ahead at the county level. They used only the current and previous incident cases and did not employ mobility cases or county demographics. Other researchers developed models to estimate COVID-19 risk at the county level [30].

The contribution of this paper is the integration of the mobility data besides COVID-19 statistics and county-level demographics to train a data-driven deep learning model to forecast the short-term spread of COVID-19 at the county level. Our work is novel because (i) we provide the first model to forecast the accumulated COVID-19 cases in two weeks. (ii) We perform a detailed analysis to study the effect of government responses on COVID-19 cases and the model’s ability to reflect the effect. (iii) We studied the effect of age demographics on the COVID-19 spread and our model’s predictions. This study design and the lessons learned from this research can also be used for outbreak detection and management of possible future pandemics.

## Dataset

**Fig. 1:**
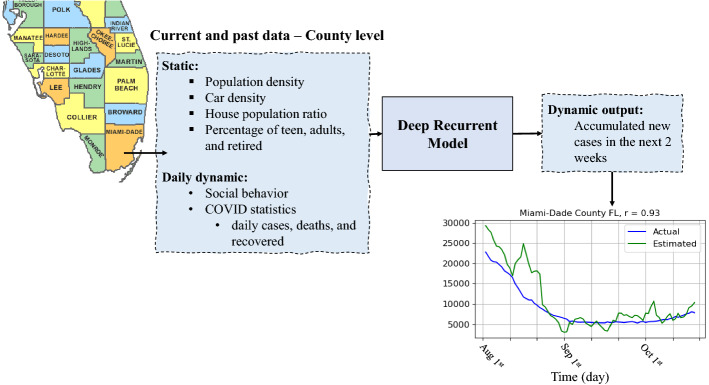
The overall diagram of the proposed method to forecast accumulated new cases in the next two weeks

The data used in our work consists of static and dynamic data at the county level as shown in Fig. 1. The static data consist of population density, household population ratio, car density, percentage of young, adults, and retirees. People age 14 to 44 were considered young, 45 to 64 adults, and 65 and older were considered retirees. The dynamic data consist of the mobility data representing the social behavior and COVID-19 statistics, including daily positive, deaths and recovered cases, and an immunity factor. The mobility data is the volume of trips people requested using the Apple Maps App relative to a baseline volume on Jan 13, 2020 [31]. The trips can be walking, driving, or bus transmission. These data are reported daily at the city level, and only driving data is reported at the county level, which is used in this study. The users’ data is associated with random identifiers when sent to the Map service and then aggregated with other users’ data at the county level, so the individual movements are not recorded. No mobility data are reported for a county when a minimum threshold of trips per day is not satisfied. The total number of counties with no mobility data from Apple was 1075 when we retrieved the data on Feb 1, 2021.

The static data and COVID-19 statistics were provided at the county level by LexisNexis Risk Solutions through High-Performance Computing Cluster (HPCC) systems [32, 33]. The static data was initially retrieved from the US Census Bureau and LexisNexis Profile Booster data source. However, the Profile Booster Aggregates data extends beyond the credit file—drawing from 45 billion public and proprietary records across more than 10,000 data sources. COVID-19 reports were daily retrieved from John Hopkins University [34]. The data were then cleaned, enhanced, and stored in HPCC Systems Data Lake as the COVID-19 statistics [33]. Additional details about this process was provided in Additional file 1: Section S1. The immunity factor is the fraction of the recovered and vaccinated population and thus considered immune.

For this study, we retrieved dynamic data from Feb 15, 2020, to Jan 22, 2021. We filtered the counties with a population density of fewer than 150 people per square mile. A total of 531 counties is included in the analysis. The data of each county has 11 attributes for 246 days. Since each data sample has low dimensionality, we did not use a dimensionality reduction method such as Principal Component Analysis [35]. There are other sources for the COVID-19 statistics, such as the COVID-19 tracking website of the NY Times [36]. Other sources for mobility data are COVID-19 community mobility provided by Google [37] and trips by distance provided by the Bureau of Transportation Statistics [38].

## Methods

We developed a deep learning model based on Long Short-Term Memory (LSTM) to forecast the accumulated number of COVID-19 cases in the next two weeks as shown in Fig. 1. LSTM is a particular type of Recurrent Neural Networks (RNNs) that has been shown to efficiently learn the temporal dependencies of time series data in many applications [8, 39]. LSTM-based algorithms are efficient in estimating influenza-like illness dynamics [40, 41]. In this study, we selected an LSTM-based model. We trained our LSTM model to learn how the past and the current number of cases and people’s mobility impact future cases. Such a model can be used to predict the accumulated number of cases in the next two weeks according to the current and past changes in COVID-19 statistics and people’s social behavior.

In our model, current data point ($\vec{d}_{t}^{{(c)}}$) at day *t* of county *c* is linearly transformed using Eq. (1) to match the number of hidden states (${N_H}$) of the LSTM network:

1 $$\vec{x}_{t}^{{(c)}} = W_{{fx}} \vec{d}_{t}^{{(c)}} + b_{{fx}}$$

where $\vec{x}_{t}^{{(c)}} \in {\mathbb {R}}^{N_H}$ and $W_{fx}$ and $\vec{b}_{fx}$ are a weight matrix and a bias vector, respectively. The output of Equation (1), $\vec{x}_{t}^{{(c)}}$, is fed to the LSTM network.

An LSTM network is built of one or more layers, where each layer has four gates of input (*i*), modulation (*g*), forget (*f*), and output (*o*), and one memory cell, $m_t$, at time step *t*. The operations in these gates are performed on $\vec{x}_{t}^{{(c)}}$ using the ${N_H}$ hidden states ($h_{t-1} \in {\mathbb {R}}^{N_H}$) and internal states ($m_{t-1} \in {\mathbb {R}}^{N_H}$) from the previous day as defined below:

2 $$\begin{aligned}&i_t=\sigma (W_{xi} \vec{x}_{t}^{{(c)}} + W_{hi} h_{t-1} +b_i) \end{aligned}$$

3 $$\begin{aligned}&g_t=\phi (W_{xg} \vec{x}_{t}^{{(c)}} + W_{hg} h_{t-1} +b_g) \end{aligned}$$

4 $$\begin{aligned}&f_t=\sigma (W_{xf} \vec{x}_{t}^{{(c)}} + W_{hf} h_{t-1} +b_f) \end{aligned}$$

5 $$\begin{aligned}&o_t=\sigma (W_{xo} \vec{x}_{t}^{{(c)}} + W_{ho} h_{t-1} +b_0) \end{aligned}$$

6 $$\begin{aligned}&m_t=f_t m_{t-1}+i_t g_t \end{aligned}$$

7 $$\begin{aligned}&h_t^{(c)}=o_t \phi (m_t) \end{aligned}$$

where $W_{ab}$ is a weight matrix ($a=\{x,h\}$ and $b=\{i,g,f,o\}$), and $\sigma$ and $\phi$ are the logistic sigmoid and tanh activation functions, respectively. The weight matrices are learnt during the training step. The current input $\vec{x}^{(c)}_t$ and previous hidden states $h_{t-1}$ are multiplied with these weight matrices then passed through the activation functions. These operations help keep relevant information from the input and update the current hidden and internal states of the LSTM.

The accumulated cases in the next two weeks (${\hat{y}}^{(c)}_{t+14}$) is calculated first by feeding the data points from day $t-T$ to day *t* ($D^{(c)}=[ {d}^{(c)}_{t-T}, {d}^{(c)}_{t-T+1},\ldots , {d}^{(c)}_t$) to a many-to-one LSTM network. Second, the hidden state, $h_{t}^{(c)}$, of the last LSTM layer is passed through two fully connected layers shown in Eq. (8) with, respectively, 512 and 1 nodes. These values were found experimentally to be suitable for our application. The first layer is followed by a ReLU activation function. The LSTM layers and the first fully connected layer are followed by a dropout layer with 0.5 drop-out rate during training to prevent overfitting. The output ${\hat{y}}^{(c)}_{t+14}$ represents the the accumulated number of COVID-19 cases in the next two weeks.

8 $$\begin{aligned} {\hat{y}}^{(c)}_{t+14}=W_{hy} h_{t}^{(c)} +b_y \end{aligned}$$

A grid search is applied to find the best number of layers (1, 2, or 3) and hidden nodes (32, 64, 128, 192, 256, or 320) based on a validation set. The model is fine-tuned weekly when more data points and the corresponding labels are available. The main reason for fine-tuning is that people’s social behavior and the governments’ regulations change over time as we learn more about the virus. As a result, new patterns appear in the COVID-19 statistics and mobility rates, which the model has to learn.

For comparison reasons, we also implement a Gradient Tree Boosting model (GTB) for COVID-19 forecasting [42]. GTB is an ensemble of multiple weak regression trees learned using an additive training strategy to learn one tree in each iteration. GTB has a comparative performance to LSTM in some applications, for example, forecasting COVID-19 cases at the country level [43–45] and biomedical time series [46]. A grid search based on a validation set is applied to find the best number of trees, the depth of each tree, and the percentage of features used per tree. In a similar fashion to train LSTM, GTB is retrained weekly when more data points and labels are available.

## Results and discussion

Most of the US states had the first wave of COVID-19 by Aug 1, 2020. Training on the rise and fall of the COVID-19 waves helps the model sufficiently learning to forecast both the incline or decline in the accumulated cases in the next two weeks. Hence, we used the data before Aug 1, 2020, to train the deep learning model. From this data, 80% of the counties were used for training. The remaining 20% were used for validation purposes to optimize the model hyperparameters (i.e., the number of layers and hidden nodes) and to select generalized model weights. The training and validation data started from Feb 15, 2020, to Jul 31, 2020. We used the data of 424 counties over 168 days for training and 107 counties over 168 days for validation. The data from Aug 1, 2020, until Jan 22, 2021, of all counties were used for testing the developed model. We used the data of 531 counties over 161 days for testing. During this period, most counties experienced their first or second wave of cases. That was why we selected that interval to evaluate the model efficacy for estimating an incline or decline in the number of cases.

Our deep learning model was implemented and trained in Keras with TensorFlow as the backend [47]. We used a computer with Windows 10 and Intel-Core-i7 CPU, 32 GB of memory, and NVIDIA-GeForce GPU with 12 GB memory for implementation purposes. The model was trained using Adam optimizer to minimize the mean squared error loss. The model performance was evaluated using two metrics: the mean absolute error (MAE) and the Pearson correlation (*r*) between the estimated and actual accumulated number of COVID-19 cases in the next two weeks as shown in Eqs. (9) and (10), respectively.

9 $$\begin{aligned} MAE = \sum _{c=1}^{C}{\sum _{t=0}^T{\frac{|{y_t}^{(c)}-\hat{y_t}^{(c)}|}{C*T}}} \end{aligned}$$

10 $$\begin{aligned} r = \sum _{c=1}^{C}{\sum _{t=0}^T{\frac{cov({y_t}^{(c)},\hat{y_t}^{(c)})}{C*T*\sigma ({y_t}^{(c)})*\sigma (\hat{y_t}^{(c)})}}} \end{aligned}$$

where C is the number of counties, T is the number of days in a giving set, $\sigma$ is the standard deviation.

### Accuracy of the COVID-19 forecasting model

The results of the proposed deep learning model are shown in Table 1. Using the mobility data, the model was able to fit the training data with a significant training and validation correlation ($\approx$ 0.8 (*p* = 0.0169)). The selected LSTM model based on the validation data has one layer and 128 hidden states. The testing correlation was also significant with (*r*) = 0.83 (*p* = 0.0053). The average testing MAE was 605.4 accumulated cases, which was higher than the validation MAE with 145.96. After our careful analysis, we noticed that the main reason for the increase in testing MAE was that during the testing interval, especially after Dec 1, 2020, there was an increase in the number of cases (i.e., many times higher) of the validation interval (Feb 15, 2020 to Jul 31, 2020). We also tested the importance of the mobility data in the successful prediction of new cases by removing the mobility data from the training-validation-testing steps. When the mobility data was excluded from the model inputs, the training and validation correlation dropped by 10% to $\approx$ 0.7 (*p* = 0.0158). The testing correlation was also significant but slightly lower (*r* = 0.82 (*p* = 0.0027)). This observation suggests that at the beginning of the pandemic, people’s mobility might be a more contributing factor to the number of cases than later when we learned more about the novel virus and how to avoid contracting the disease by wearing masks etc.

**Table 1:** The results of the proposed approach to forecast the accumulated cases in two weeks

Using mobility data	Training		Validation		Testing	
	MAE	Correlation (p)	MAE	Correlation (p)	MAE	Correlation (p)
Yes	169.62	0.78 (p = 0.0169)	145.96	0.79 (p = 0.0083	605.40	0.83 (p = 0.0053)
No	182.14	0.68 (p = 0.0158)	150.91	0.69 (p = 0.0114)	596.66	0.82 (p = 0.0027)

**Fig. 2:**
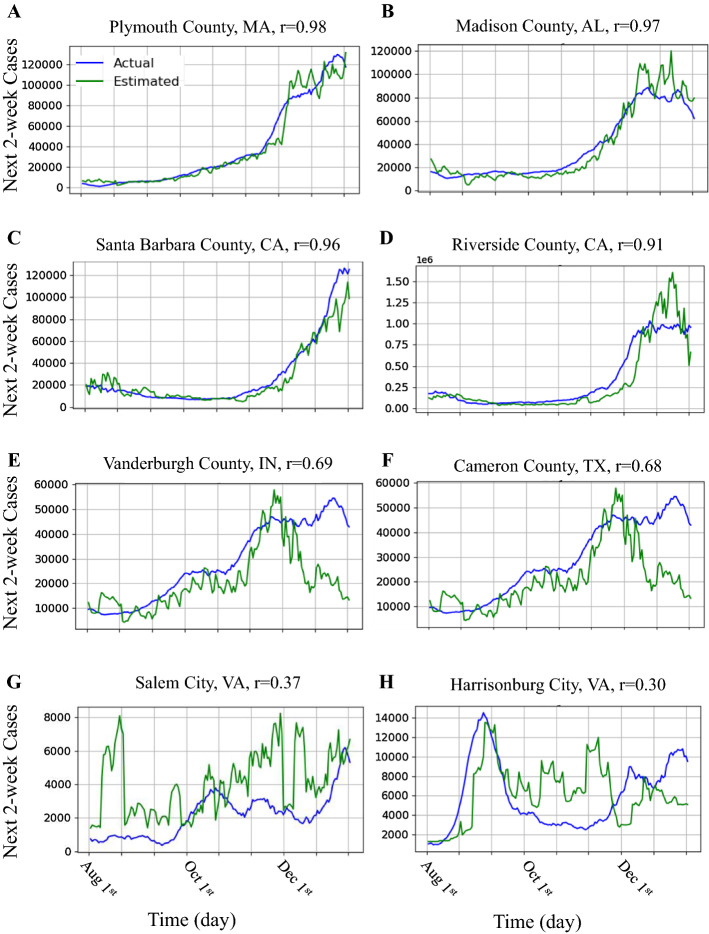
The estimate and actual accumulated COVID-19 cases for eight counties are provided. **A**–**D** show accumulated cases of counties for which our model provides a high correlation > 0.9. A and C show counties with an increase in the number of cases v.s. B and D show a decrease in the number of cases. **E**–**H** shows accumulated cases of counties for which our model provides a moderate and low correlation

Eight samples of our model predictions and the actual accumulated cases during the testing interval are shown in Fig. 2. Plots in A-D show counties for which our model provided a high correlation of >0.9. Plots in E-F show counties with a moderate and G-H a low correlation. It is interesting to observe that the model provided early detection of the outbreak in A-F counties. It is also interesting that the model predicted a decrease in the number of cases in counties B and D.

### Comparison with gradient tree boosting model

The GTB was implemented using the XGboost library in Python and trained using a 0.1 learning rate. The selected GTB model based on the minimum validation loss had 130 regression trees, a maximum depth of 5 leaves, and 40% of features per tree. The GTB model fitted the training data with a training and validation correlation of 0.79 and 0.76, respectively. Training MAE was 156.6, and validation MAE was 183.6. The testing correlation was 0.67 (*p* = 0.01), and the testing MAE was 883.9 accumulated cases, which indicated that the LSTM outperformed the GTB.

### Correlation analysis for individual counties

**Fig. 3:**
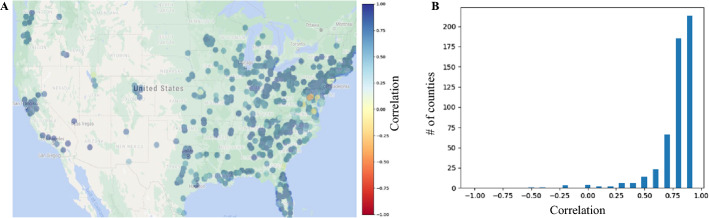
Correlation between the predicted and actual accumulated COVID-19 cases in the testing data. **A** Spatial distribution of the testing correlation per county. **B** Histogram of the correlation for the US counties

Figure 3A shows the spatial map of the testing correlation for each county on the US map. As indicated by the blue color on this spatial map, our model successfully predicted the total COVID cases. To further confirm this observation, we show the number of counties with a specific range of correlation in Fig. 3B shows. It can be seen that the majority of the counties (i.e., 87%) had a correlation of > 0.7 across the states.

### Relationship between model performance and number of cases

**Fig. 4:**
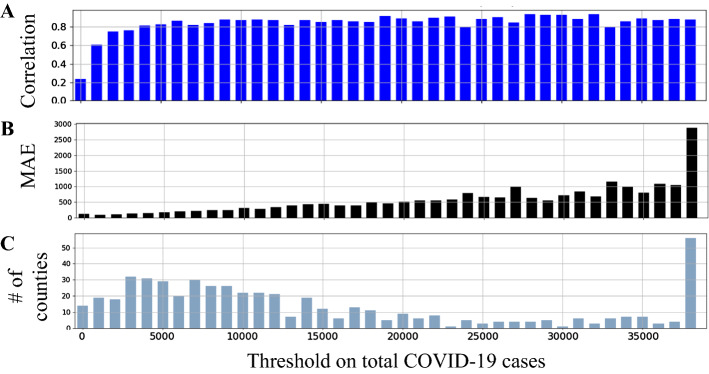
The model performance for counties with specific ranges of total cases during the testing interval. The correlation and MAE are shown in part A and B. The number of counties for each range of total cases is shown in part C. The last bar represents the counties with total cases of more than 40,000

We investigated whether the number of cases in a county affects the forecasting ability of the model. For this purpose, we set a threshold on the minimum number of COVID-19 cases in the counties and provided the correlation and MAE metrics for the selected counties Fig. 4. It provides the averaged performance metrics and the number of counties for a threshold ranging from 0 total cases to 40,000. As we can see, the correlation between the predicted and actual number of cases did not change significantly with the number of cases. The correlation was about 0.8 for most counties except for the counties with less than 2000 total cases after Aug 1st, 2020. As expected, the MAE was higher as the number of total COVID-19 cases was more.

### Ability to reflect changes in COVID-19 cases due to government response

**Fig. 5:**
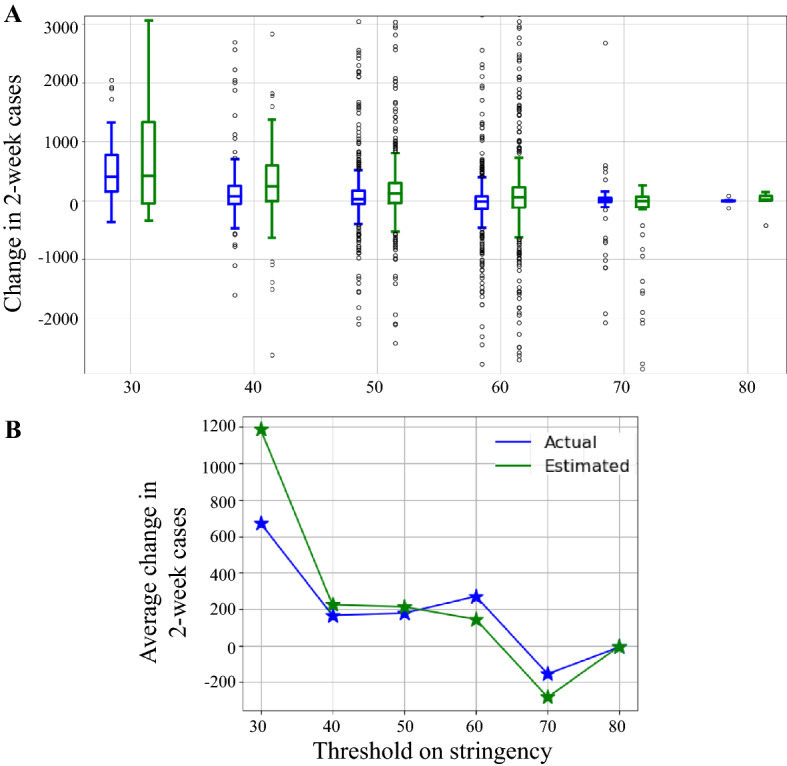
The change in accumulated daily cases for two weeks as estimated by the model and the actual cases 2 weeks after a change in the stringency level. **A** shows the change box plots for all counties during the testing interval for each stringency range, and **B** shows the averaged changes

Change in lockdown policies, mask mandates, and other government responses directly impact the daily COVID-19 cases. Hence, the model predictions of the 2-week daily cases have to reflect that impact as shown by the actual accumulated 2-week cases. To analyze the model’s ability to demonstrate the effect of policy changes, we utilized the government responses provided by Oxford COVID-19 Government Response Tracker (OxCGRT) [48]. From OxCGRT, we used a stringency index which is an average of the indicators of closures and containment and public info campaigns. This index is between 0 (no restrictions) and 100 (stringent restrictions) and is reported daily at the county level. Indicators of closures and containment include closing schools, workplaces, public transportation; cancellation of public events; restrictions on gatherings; staying at home requirements; and restrictions on internal movements and international travels. We found the effect of the stringency index on the actual and estimated cases by considering one month after any changes in stringency-index levels. We considered 10 levels (0–10, 11–20, ..., 91–100). The change in 2-week cases was calculated as the accumulated cases in the last two weeks minus accumulated cases in the first two weeks of the month following the changes in stringency levels. During this month, the accumulated cases of the first 2 weeks are due to the effect of the previous policy, and the accumulated cases of the last two weeks are due to the effect of the current policy. Figure 5A shows the box plots of changes in the 2-week cases during the testing interval for each stringency range at the county level. Figure 5B shows the averaged changes for each stringency range. These plots show both actual changes and the predicted changes based on the developed model with the mobility data. As we can observe from these plots, a higher stringency index decreased the cases. The stringency index was used as an external feature that we did not was feed to the model. The choice of not including the stringency index was not to bias the analysis of the model’s ability to capture change in COVID-19 cases due to government responses.

It is important for the developed model to reflect a similar behavior as the actual total cases with the changes in the government policy. The predicted average change in 2-week cases closely follows the actual average change, as shown in Fig. 5B. The worst-case scenario in the number of cases was for 30-stringency level when there was about 700 average increase in the cases after reducing the stringency level. For stringency levels, 40–60, both the predicted and actual changes show about 200 average increase in the case numbers. For stringency levels of 40–60, both the predicted and actual changes show about 200 average increase in the case numbers. For the stringency levels 70 and 80, both the predicted and actual changes show about 200 average decrease or the same number of cases. Please note that there were only 9 cases with the 80-stringency level. To further validate our observation, we applied the paired t-test on the predicted and actual changes in 2-week cases at each stringency level. The null hypothesis is that the predicted and actual changes have identical average values. The null hypothesis held true for all stringency levels with *p* > 0.05 except for the 30-stringency level (*p* = 0.038).

### Ability to capture the effect of age demographics on COVID-19 cases

**Fig. 6:**
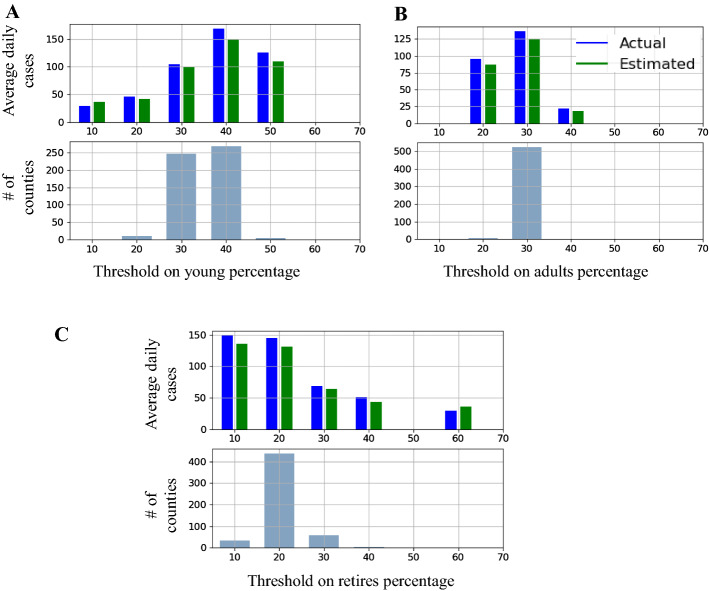
The actual and the predicated average daily cases during testing interval distributed based on the percentage of young, adult, and retiree people in each county. The trend of the average daily cases based on young, adult, and retiree demographics are shown in **A**, **B**, and **C**, respectively

Figure 6 analyzes the effect of age demographics on the average daily cases. Specifically, we looked into three age demographics of young, adults, and retirees. For each age population, we identified the counties with people greater than a percentage. For example, we identified counties where 10% of their population is young and calculated the average daily cases. We increased the threshold by 10% until 70% and repeated the analysis. The number of counties and the average daily cases of actual and predicted data were shown in Fig. 6A. A similar analysis was performed for the adult and retiree population as shown in Fig. 6B and C, respectively.

As can been from these plots, the average daily cases doubled when the young population increased from 10 to 20% and tripled when increased to 30%. The inverse pattern happens with the increase in the percentage of retires. Our model was also able to capture the effect of age demographics on the COVID-19 spread. Average daily cases decrease with an increase in the retiree percentage and increase with the young population percentage increase. The summary of the paper findings is reported in Table 2.

**Table 2:** Summary of the paper findings

Accuracy of the COVID-19 forecasting model	Significant testing correlation 0f 0.83 (p = 0.0053) with MAE of 605.4 accumulated cases
Correlation analysis for individual counties	The majority of the counties had a correlation of>0.7 across the states
Relationship between model performance and number of cases	The county number of cases did not affect the model performance.
Capturing change in COVID-19 cases due to government responses	The model captures the decrease in the cases with higher stringency index
Ability to capture the effect of age demographics on COVID-19 cases	Average daily cases has reverse proportionality with the retire percentage and direct proportionality with the young population percentage

### Comparison to related work

To the best of our knowledge, few studies were published to forecast daily or weekly incident cases of COVID-19 at the US state or county level [22, 28, 29]. Prior research performed the forecasting at different spacial resolution (e.g. states or counties) and different temporal intervals (e.g. daily or weekly), and evaluated at different period of time. These differences make a direct comparison not applicable. For example, Change et al. developed a SEIR model for ten of the largest US metropolitan areas where COVID-19 and hourly cell-phone mobility data were integrated to track visits to points of interest [22]. They fitted their model on the data from March 8 to April 15, 2020, and reported a 406 root mean square error in estimating daily cases for Chicago between April 15 to May 9, 2020. We used the data during this period for validation purposes in our work, and we reported a significantly lower MAE (145.96) at the county level. The MAE was higher during the testing duration due to the high increase in the actual number of cases compared to April-May 2020. Kapoor et al. developed a Graph Neural Network to forecast next-day COVID-19 cases [28]. Their network performs lower than a Recurrent Neural Network when estimating the change in daily cases in 20 US counties. We could not directly compare with their results since they estimated the following day cases, which would also challenge the applicability of such a prediction for use in policy changes.

Adiga et al. [29] developed a Bayesian ensemble of various models (e.g., Auto-Regressive, SEIR, LSTM, etc.) to forecast the weekly accumulated cases 1 to 4 weeks ahead at the county level. They used only the current and previous incident cases and did not employ mobility cases or county demographics. For the 2-week ahead forecast starting August 2020 to January 2021, they reported an MAE of about 125 when using the Bayesian ensemble of all the models. Removing any of the models resulted in a significant increase in MAE to over 900. Their MAE when using the ensemble of all models was better than our LSTM model, but removing any model from their ensemble resulted in worse MAE than ours. They considered all the counties in the US, including the counties with a low population density, which affects its comparison to our method since we considered only counties with a high population density. Counties with low population density have a lower number of cases and thus lower MAE in general, as shown in Fig. 4B. Therefore, MAE averaged across all counties is lower than MAE averaged only for high population density. Also, we accumulated the cases for two weeks then evaluated the model, whereas they accumulated the cases weekly in their work. Besides the previous publications, the COVID-19 Forecast Hub contains several models to forecast mortality and incident cases at the nation and state levels [27]. As of March, 30 2021, no evaluation of county-level forecast has been reported at the hub [49].

### Study limitation

Our deep learning model successfully forecasted the new cases in two weeks; however, its performance could be significantly improved by incorporating government regulations such as mask mandates or people’s adherence to the pandemic-related regulations. We did not have access to such data, so we could not include it in our models. Another limitation is that deep learning models learn only from the patterns exhibited in the training data; thus, any new lockdown measures that had not been implemented before may impact the model estimation for the future accumulated cases. However, this limitation is partly solved by fine-tuning the deep learning model weekly.

## Conclusions

We developed a deep recurrent model based on LSTM to forecast the accumulated number of COVID-19 cases at the county level across the US. Our model receives the counties’ demographics and previous daily social behavior and COVID-19 statistics and predicts the total COVID-19 cases in two weeks. The model resulted in a significant correlation when tested on the interval from Aug 1, 2020, until Jan 22, 2021. It was able to predict an increase and also a decrease in the total number of cases. We performed a detailed analysis to validate that the predictions from our model reflect the same patterns in the actual cases with respect to the changes in the government pandemic regulations and counties’ age demographics. In sum, our analysis showed that our model has the potential to predict an outbreak in COVID-19 cases two weeks in advance. Such a model is specifically important in the COVID-19 pandemic. Many infected populations remain asymptomatic while spreading the virus, making it challenging for traditional mechanistic models to predict an upcoming outbreak accurately. Our work has a significant application for effective management of the pandemic and future outbreaks and could potentially help to save lives and restart the economy quickly and safely.

## Supplementary Information

**Additional file 1.** Processing of COVID-19 daily reports retrieved from John Hopkins University.

## Data Availability

The used data is available publicly on Apple Inc., US Census Bureau, and LexisNexis Risk Solutions. The code used for this study are made publicly available on https://github.com/Murtadha44/covid-19-spread-risk.

## Abbreviations

COVID-19: Coronavirus disease 2019
RNN: Recurrent neural network
LSTM: Long short-term memory
*r*: Pearson correlation
MAE: Mean absolute error
N_H_: Number of hidden states
W_ab_: Weight matrix (a = {x, h} and b = {i, g, f , o})
OxCGRT: Oxford COVID-19 Government Response Tracker
HPCC: High-performance computing cluster
