# Supplementary material for: The forecast of COVID-19 spread risk at the county level
Source: J Big Data. 2021 Jul 7;8(1):99. doi: 10.1186/s40537-021-00491-1 (PMC8261401; doi:10.1186/s40537-021-00491-1)
Supplement: Supplementary file 1 — Additional file 1. Processing of COVID-19 daily reports retrieved from John Hopkins University. [file 40537_2021_491_MOESM1_ESM.docx]

**Additional file Online Content for “The Forecast of COVID-19 Spread Risk at The County Level”**

**S1. Processing of COVID-19 daily reports retrieved from John Hopkins University**

COVID-19 reports were daily retrieved from John Hopkins University (JH) [1]. The data were then cleaned, enhanced, and stored in HPCC Systems Data Lake as the COVID-19 statistics. The processing steps are as follow:

1. Upload the JH data from their site via FTP
2. Clean up FIPS codes so that they are all five digits with leading zeros.  Strip out any decimal points.  The source data has inconsistent formatting of FIPS which would result in bad JOINs.
3. Many areas changed the granularity of their reporting locations from time to time.  We retain a map of these changes so that we can calculate a consistent time series.  Otherwise, we end up with time-series with no new data, and others that begin late.  We map to the coarsest location during the life of the data series.  For example, several NY Boroughs were originally mapped to New York City.  Then the individual Burroughs started reporting and the NYC time series ended.  We continue to map those Burroughs to New York City, sacrificing the new granularity for time series consistency.
4. Compensate for JH Format change after 3/22/2020, by normalizing to a common format.
5. Clean up some broken location hierarchies, particularly UK and FRANCE.  Some data is reported at Level 3 (state/ provinces level) with no Level 2 (county level) specified.
6. Join the population data for each location with the JH COVID-19 data.
7. Join the vaccination data for each location with the JH COVID-19 data / population data
8. Some data is reported at Level 3, some at Level 2, and some at Level 1.  We roll up the Level 3 data to Level 2, and the Level 2 data to Level 3, so that e.g. all states / provinces are shown at level 2, even though some states may have reported at Level 2 and some at Level 3.  All countries are visible at Level 1, even though some may have reported at Level 2 or Level 3.  Accumulation is done by hierarchical aggregation of raw (cumulative) data.
9. Apply filtering in order to minimize the effect of anachronistic updates.  These occur when data is adjusted on a date that was not when the data actually occurred.  Examples:  Missing data added, After-the fact adjustments made.  These changes are common in the raw dataset, and can result in cumulative values going backward, or many thousands of cases being reported in a single day that did not all occur on that day.  This makes the time series useless for most analysis, as it implies negative or unrealistically high R values.  The filter removes most of these effects without delaying the time series.  It is better to ignore adjustments since we are mostly concerned with differential statistics, not absolute values.  We also maintain the absolute values so that our reporting of e.g. Total Cases to Date will match other sources.  These totals are not used for analytics however.
10. Calculate a series of daily statistics including infection state, and surge detection.
11. Calculate a series of periodic (weekly by default) metrics.
